# Benzoxaboroles—Novel Autotaxin Inhibitors

**DOI:** 10.3390/molecules24193419

**Published:** 2019-09-20

**Authors:** Kristina Kraljić, Dubravko Jelić, Dinko Žiher, Adam Cvrtila, Snježana Dragojević, Verona Sinković, Milan Mesić

**Affiliations:** Fidelta Ltd., Prilaz baruna Filipovića 29, 10000 Zagreb, Croatia; kristina.kraljic@glpg.com (K.K.); dubravko.jelic@glpg.com (D.J.); dinko.ziher@glpg.com (D.Ž.); adam.cvrtila@glpg.com (A.C.); snjezana.dragojevic@glpg.com (S.D.); verona.sinkovic@glpg.com (V.S.)

**Keywords:** Autotaxin (ATX), lysophosphaditic acid, benzoxaboroles, cancer

## Abstract

Autotaxin (ATX) is an extracellular enzyme that hydrolyses lysophosphatidylcholine (LPC) to lysophosphatidic acid (LPA), which has a role in the mediation of inflammation, fibrosis and cancer. ATX is a drug target that has been the focus of many research groups during the last ten years. To date, only one molecule, Ziritaxestat (GLPG1690) has entered the clinic; it is currently in Phase 3 clinical trials for idiopathic pulmonary fibrosis. Other small molecules, with different binding modes, have been investigated as ATX inhibitors for cancer including compounds possessing a boronic acid motif such as HA155. In this work, we targeted new, improved inhibitors of ATX that mimic the important interactions of boronic acid using a benzoxaborole motif as the acidic warhead. Furthermore, we aimed to improve the plasma stability of the new compounds by using a more stable core spacer than that embedded in HA155. Compounds were synthesized, evaluated for their ATX inhibitory activity and ADME properties in vitro, culminating in a new benzoxaborole compound, 37, which retains the ATX inhibition activity of HA155 but has improved ADME properties (plasma protein binding, good kinetic solubility and rat/human plasma stability).

## 1. Introduction

**Figure 1:**
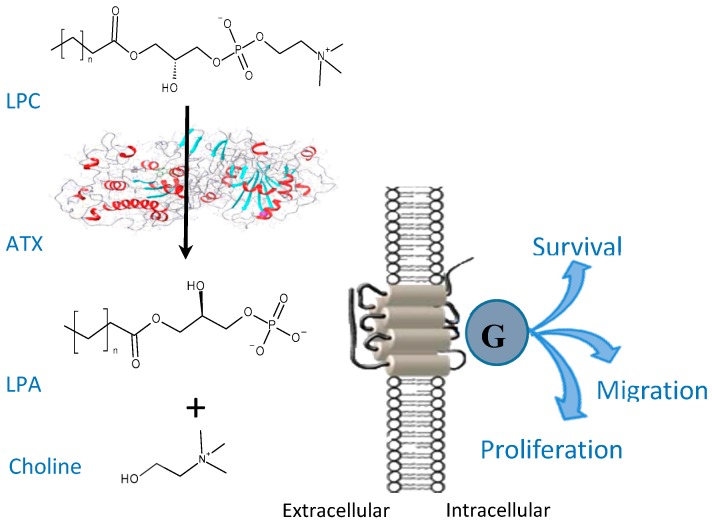
Lysophosphatidylcholine (LPC) is hydrolyzed by autotaxin (ATX) to lysophosphatidic acid (LPA); role of LPA on downstream signaling in cells.

Autotaxin (ATX) is a lysophospholipase D enzyme that hydrolyses the bioactive lipid molecule lysophosphatidylcholine (LPC) to form lysophosphatidic acid (LPA) and choline (Figure 1) [1]. ATX is present in cerebrospinal fluid, blood, peritoneal fluid and synovial fluid [2].

LPA is a mediator of several pathophysiological processes, such as inflammation, fibrosis and cancer [3]. LPA modulates six different G protein-coupled receptors (GPCRs) in the cell membrane. It has been reported that LPA has pro-tumorigenic effects on ovarian, breast and prostate cancer cells [4].

High levels of LPA have also been found in the peritoneal liquids (ascites) of women suffering from ovarian cancer [5]. High levels of LPA (up to 10 times higher than normal) have generally been observed in cancer patients compared to healthy subjects [6]. In mice, Nagano reported that a 4 mg/kg i.v. injection of an ATX inhibitor (such as 3BoA) significantly decreases LPA plasma levels (almost to zero), indicating that ATX is the major enzyme responsible for LPA production [7].

**Figure 2:**
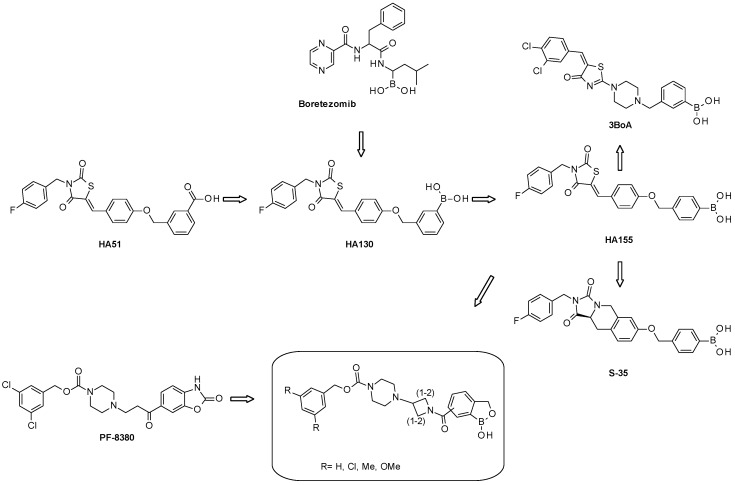
Design and development of small molecule ATX inhibitors.

In the search for non-lipid like ATX inhibitors, a thiazolidinedione carboxylic acid analog HA51 (Figure 2) has been reported [8]. Inspired by the proteasome inhibitor bortezomib, in which the boronic acid group interacts with a *N*-terminal threonine oxygen nucleophile in the proteasome, the carboxylic group in HA51 (Figure 2) was similarly replaced by the boronic acid isostere. The derived compound HA130 has reported activity of 28 nM in an in vitro LPC choline release assay. Crystallography studies published by Hausman [9] further supported the hypothesis that HA130 is engaged in interactions with Thr209 residue and that the 4-fluorobenzyl moiety reaches deep in a hydrophobic pocket.

The molecule HA130 is a potent inhibitor of ATX. Furthermore, in vivo proof of principle studies have demonstrated a rapid decrease of LPA levels in the plasma of mice after an intravenous administration (1 nmol/g), with no overt toxicity issues observed [8]. HA130, in a dose-dependent manner, inhibited the ATX-mediated migration of A2058 human melanoma cells. However, the stability of HA130 in vivo was very poor (half-life less than five minutes) [10]. A later publication from the same authors [11] reported on HA155, a boronic acid regioisomer of HA130, which has a better inhibition of ATX. The same was observed for HA130, as the presence of a double bond attached to the thiazolidinedione heterocycle may cause HA155 to act as a Michael acceptor and may cause in vivo instability. Therefore, in an effort to improve stability, a reduction of the double bond in HA155, and the introduction of the rigid bicyclic tetrahydroisoquinoline as a central ring core have been performed [11].

More recent developments around boronic acid-based ATX inhibitors were published in 2013 by Nagano’s group [7], in which thiazolinone analogues bearing benzylboronic acid groups were investigated. The boronic acid warhead, as in HA155, was designed to bind to Zn^2+^ ions and to Thr209. Changes designed to optimize lipophilic interactions with the active site of ATX, afforded derivative 3BoA (Figure 2) with good inhibitory properties (IC_50_ = 13 nM). Compound 3BoA was further tested in vitro and in vivo, showing several very promising pharmacologically related features. In brief 3BoA is more potent than HA155 as an LPA production inhibitor in incubated plasma and cell-motility assays, and it exerts a more favorable metabolic stability and decreases rapidly plasma LPA levels in mice after a 4 mg/kg intravenous administration [10]. 

A recent (2017) publication by Lanier et al. [12] reported a fragment based approach used to explore phenylboronic acids as warheads towards ATX inhibitors. They tested more than 650 boronic acid fragments. combining in silico computational chemistry filters and crystallography, allowing them to identify fragments that were consistent to known SAR against ATX [12].

The non-boronic acid compound, PF-8380, is one of the most potent in vitro inhibitors of ATX (Figure 2) with an IC_50_ = of 2.8 nM in a human enzyme assay. PF-8380 was developed by Pfizer in the context of identifying new anti-inflammatory drugs indicating promising in vivo efficacy. Notably, in a separate study, PF-8380 produced a significant anti-invasive effect in glioblastoma cell lines, delaying simultaneously glioma tumor growth progression in vivo [8].

**Figure 3:**
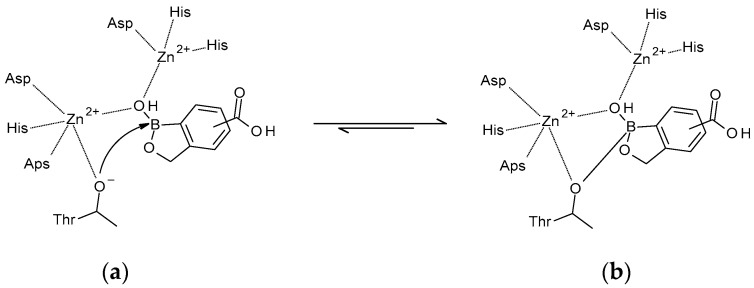
Formation of an adduct of benzoxaborole in the ATX active site according to literature [12]. (**a**) Nucleophilic attack on benzoxaboroles by catalytic threonine oxygen side chain; (**b**) reversible covalent tetrahedral adduct.

The Ovaa group has speculated that replacing carboxylic acid warheads with less acidic boronic acid warheads may be beneficial for ATX inhibition. Benzoxaboroles, (cyclic boronic acid esters that have lower pKa values that are 1–2 units lower than boronic acids [13]) may have improved binding characteristics towards the ATX enzyme. Crystallographic studies by Hausman have demonstrated further insight for the boronic acid binding mode, in which Thr209 and Zinc atoms are in a complex with boronic acids [9]. This was illustrated by the ring strain generated in the five-membered oxaborole ring where the boron atom was in a neutral, trigonal-planar form [13]. Benzoxaboroles have physicochemical properties which are a consequence of the relatively strong Lewis acidic center on the boron atom and the presence of a free hydroxyl group [14]. The *sp^2^* hybridized boron atom possesses an empty p-orbital which accepts electrons from the hydroxyl group of threonine 209 (Thr209) that explains adduct formation of boronic acids, as in the recent modelling of Lanier [12]. According to Lanier et al. [12], a boronic acid motif with this type of interaction may enhance binding affinity up to 1000 fold. Similarly, we assumed that the binding mode and the mechanism of adduct formation in the ATX active site would resemble phenylboronic acids (Figure 3).

Our group was interested in new ATX inhibitors for cancer treatment. Based on the previous work [9] where boronic acids were used as warheads, we designed and synthesized novel ATX inhibitors with a benzoxaborole as an isostere aiming for the same binding pattern (like HA155) to the active pocket but with better overall drug properties. Working around the potential stability issue due to presence of the double bond, we designed different linkers, introducing more rigidity. We also used the 3,5-substituted benzylic group as a hydrophobic lipid binding motif.

**Figure 4:**
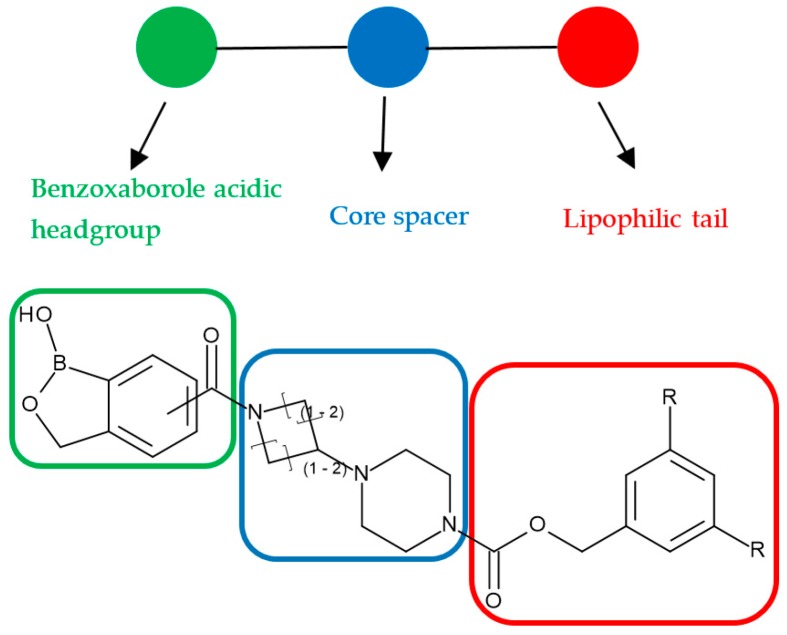
General structure of novel ATX inhibitors.

Our design and synthesis of novel ATX inhibitors focused on inhibitors with the benzoxaborole head moiety as the acidic headgroup, the substituted benzyl carbamate moiety as the lipophilic portion, and a rigid core spacer constituted of two saturated heterocyclic rings (Figure 4).

**Figure 5:**
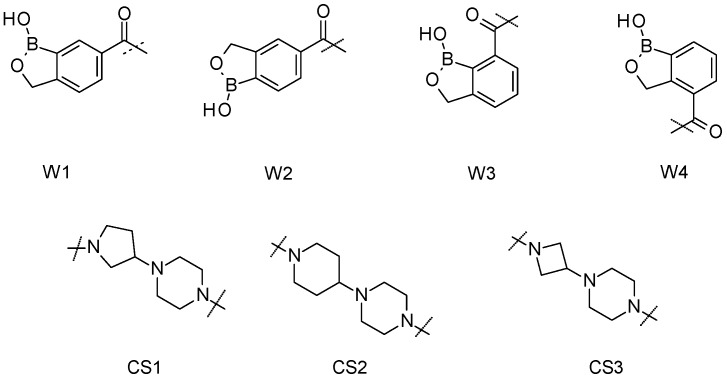
Structures of the warheads (W) and the core spacers (CS).

The structures of the warheads and spacer groups to be combined together are shown in Figure 5.

**Figure 6:**
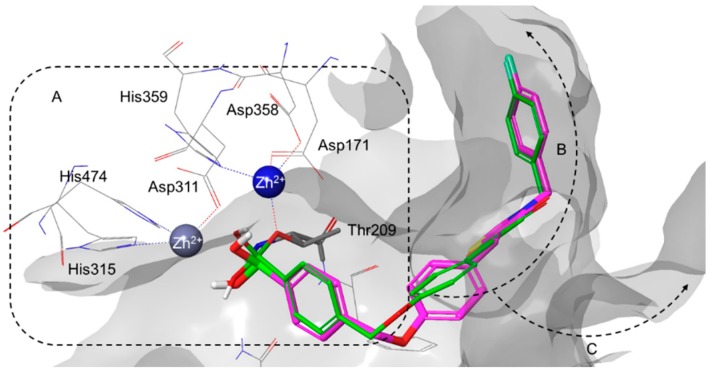
Overlay of **HA155** boronic acid (green) inhibitor in the binding site of ATX (Protein Data Bank (PDB) ID: 2XRG [9] and re-docked pose of **HA155** (magenta) using Covalent Docking v1.3. [15].

The design of the novel ATX inhibitors was also based on the available protein-ligand x-ray crystal structures in the PDB (Protein Data Bank) (Figure 6).

**Figure 7:**
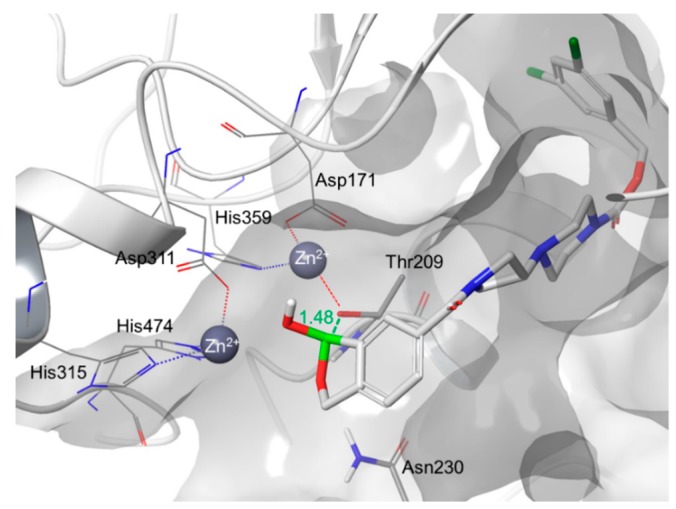
Compound **37** docked in the binding site of ATX using covalent docking. The covalent bond between the oxygen atom of Thr209 and the boron atom of boronate is labelled in green (d(O–B) = 1.48 Å).

**Figure 8:**
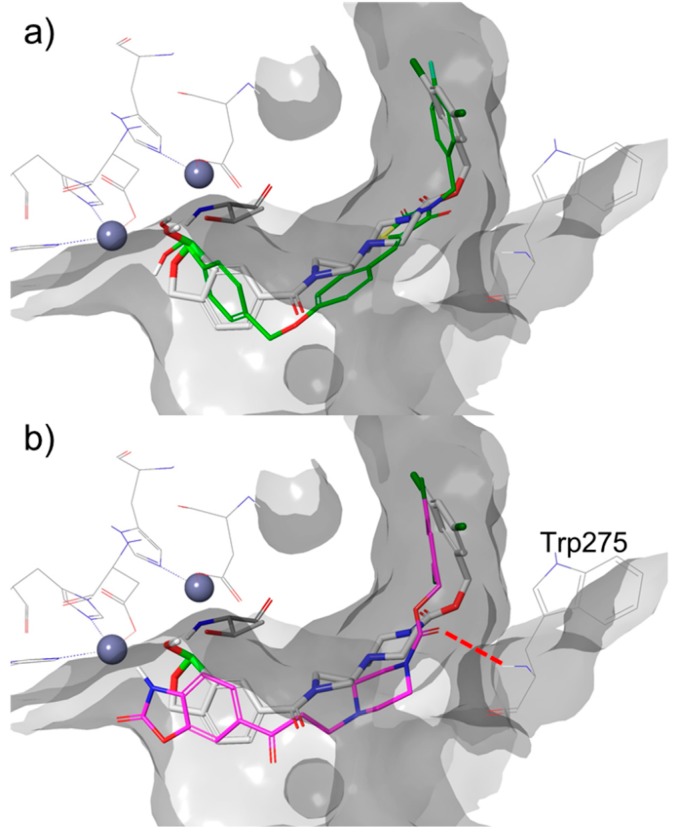
Compound 37 docked in the binding site of ATX using covalent docking and overlapped with ligands from x-ray structures: (**a**) HA155 boronic acid inhibitor (green, PDB ID: 2XRG) [9]; (**b**) PF-8380 (magenta, PDB ID: 5L0K [21]).

ATX inhibitors [16] (Figure 7) target the hydrophobic lipid-binding pocket in the central catalytic phosphodiesterase (PDE) domain of ATX (Figure 8A). This is located underneath a shallow groove that accommodates lysophospholipids (Figure 8B). In the active site, the first Zn^2+^ ion is in the tetrahedral arrangement [17] and is coordinated by the catalytic Thr209 as well as the side chains of Asp171, Asp358, and His359. The second Zn^2+^ ion coordinates with Asp311, His315 and His474, and, usually, a solvent molecule or counter-ion. Furthermore, an open tunnel (or ‘channel’; Figure 6C), which is partially hydrophobic in nature, is located in close proximity where a variety of molecules can be accommodated, forming a ‘T-intersection’ with the shallow groove [18,19]. 

In our hands, a standard docking protocol within Glide (with and without H-bond/metal constraints) failed to dock HA155 in the active site of ATX in the pose observed in the x-ray structure (PDB ID: 2XRG [9]) This pose has the characteristic interaction of Thr209 with the boron atom. Based on natural bond orbital (NBO) calculations [20], the existence of polar character of Thr209 and boron atom of HA155 hybridized to *sp*^3^ character, we applied a covalent docking approach. Using covalent docking, HA155 was successfully docked in the conformation observed in the crystal structure (RMSD (heavy atoms) = 0.68 Å, Figure 6). This same approach was therefore applied to our compounds in this paper. (Figure 7).

The dative covalent bond between the boron atom and Thr209 is required for these inhibitors [11], and docking poses thus generated are in line with the previously reported co-crystalized structures of ATX (Figure 8a). In addition, this reversible covalent bond places the hydroxyl moiety of the boronate between both zinc ions in the ATX active site (Figure 7). The docking studies also showed that the other oxygen atom of the boronate is involved in an interactions with the amides of Thr209 (d(O…H–NThr209) = 2.6 Å) and/or Asn230 Thr209 (d(O…H–NAsn230) = 2.3 Å), which are in close proximity. The binding of the rest of the molecule is mainly driven by hydrophobic interactions in similar way as other ligands with similar ‘tails’—such as PF-8380 (PDB ID: 5L0K [21], Figure 8b). The aromatic ring at the end of the ‘tail,’ a hydrophobic 3,5-dichlorobenzene, points directly into a deep hydrophobic pocket. The hydrogen bond between the carbonyl group pf the carbamate and the Trp 275 amide has been observed in the generated docking poses for some compounds described in this paper, similar to what is revealed in the co-crystalized structure with PF-8380 (d (O…H–NTrp275) = 3.1 Å, Figure 8b) and ATX.

As previously suggested for SAR transfer between series of active compounds, targeting this hydrogen bond can be useful in the modification of this class of benzoxaboroles [22]. The lipophilic tail of the molecule was designed according to data published for the PF-8380 molecule. This is where the carbonyl group creates a hydrogen bond with the acceptor of a Trp275 [9]. To reduce the degrees of conformational freedom, the rigidity of the molecule increased by the introduction of several acyclic rings. These rings would bridge the between warhead benzoxaborole and carbamate moiety (Figure 8).

During the final preparation of the manuscript, the Kang group analyzed the topological water network in the binding pocket of ATX. Using this pharmacophoric insight, new molecules were synthesized and tested for their ATX inhibitory activities [23].

## 2. Results

### 2.1. ATX Inhibition

All 21 new benzoxaboroles derivatives prepared herein were evaluated for ATX inhibition in vitro using a biochemical choline detection assay, combining elements of assays already described in the literature [24]. ATX activity was measured using Lyso PC (16:1) as a substrate and HA155 was used as the standard, control, compound.

clogP values were calculated for all compounds using the Percepta software [25]. Chrom logD was determined experimentally using a procedure described in the supplementary data. Furthermore, the correlation between calculated clogP values and experimentally determined Chrom log D values were explored for molecules containing boron functional groups such as boronic acids and benzoxaboroles. The results are presented in Table 1.

### 2.2. ADME Properties

Eight of the most active compounds, together with **HA155**, were selected for characterization of in vitro ADME properties such as solubility, microsomal metabolic stability, plasma stability and plasma protein binding (PPB). Results are presented in Table 2.

## 3. Discussion

The modification of several regions had dramatic effects on the inhibition of ATX. Thus, the hydrophobic part of the inhibitors showed sensitivity to phenyl substitution—the unsubstituted phenyl compound (41) being almost inactive (23 µM). The 3,5 dimethoxyphenyl analogue (49) was a slightly better (11 µM). Ten times better potency was achieved when comparing to 41 with 3,5-dimethyl substitution on phenyl ring for compound 45. The original 3,5-dichlorosubstitution (already described for PF-8380) remained the most favorable substitution pattern for the majority of the compounds prepared. SAR around the core spacer showed that the piperazine–azetidine bicycle was the best spacer. The position of the boron atom on the aromatic ring (acidic headgroup) was also very important and differed to that observed with HA155. In our case, the most active compound was the 6-amido substituted benzoxaborole (compound 37), whilst 4-amido or 7-amido substitution was not tolerated. Among 21 tested compounds, four compounds showed ATX inhibition in the nanomolar range of activity. The IC_50_ for the most active compound (37) was 130 nM. In our LPC choline released assay, HA155 also showed nanomolar activity (88 nM) but was less potent then reported in published data (5.7 nM) [11]. Keeping in mind that the analogue of HA155 with tetrahydroisoquinoline as a rigid core spacer was significantly less potent (ten times) then HA155, our molecule 36 with space core rigidity was only two times less potent that HA155.

The kinetic solubility in plasma bovine serum (PBS) at pH 7.4, determined by turbidimetric method, was >100 µM for the majority of tested compounds. The most active compound 36 had a solubility of 30–100 µM, while HA155 suffered from very poor solubility (1–3 µM). Results regarding metabolic stability in rat and human liver microsomes suggest that the majority of tested compounds would be classified a high-predicted in vivo hepatic clearance (expressed as percentage of liver blood flow, %LBF). Two exceptions include compounds 37 and 45, which had a moderate predicted in vivo clearance (62% and 45%, respectively) in rats. However, in human liver microsomes, all compounds showed high-predicted in vivo clearance, whereas the comparator compound showed a moderate predicted in vivo clearance. The results of plasma stability which were measured at four hours indicated a good stability of compound 37 that remained 71.2% of its initial concentration in rat and 79.1% of its initial concentration in human plasma. However, HA155 was less stable with only 65.7% of its initial concentration in rat and 42.9% of its initial concentration in rat and human plasma, respectively, remaining after four hours. The binding to plasma proteins (PPB) of newly synthesized compounds was significantly lower when compared with binding of standard compounds that showed a very high PPB of >99.9% in both tested species. That would impact efficacy of 37 in comparison to HA155 since their free fractions are different more than 20 times assuming that both molecules have the same binding kinetics k_on_/k_off_.

## 4. Experimental Section

### 4.1. Materials and Instruments

All chemicals, solvents, and chemical and biochemical reagents were of analytical grade and purchased from commercial sources (Merck, Merck KGaA, Darmstadt, Germany, Fluka Sigma-Aldrich Laborchemikalien GmbH, Hannover, Germany, Alfa Aesar, Karlsruhe, Germany and Sigma, St. Louis, MO, USA, Combi-blocks, Combi-blocks Inc.San Diego, USA, TCI, TCI-Europe, Zwijndrecht, Belgium, Kemika, Kemika d.d., Zagreb, Croatia). All starting materials were obtained from commercial sources (Merck, Merck KGaA, Darmstadt, Germany, Fluka Sigma-Aldrich Laborchemikalien GmbH, Hannover, Germany, Alfa Aesar, Karlsruhe, Germany, and Sigma, St. Louis, MO, USA) and used without further purification.

The progress of all reactions was checked by UPLC-MS/UV Waters system (Waters, Waters Corporation, Milford, USA) and thin layer chromatography (TLC) using precoated Silica Gel 60F254 sheets (Merck, Darmstadt, Germany). The spots on plates were visualized under UV light (254 nm).

Column chromatography was performed by an Interchim Puriflash 450 system or by the Waters Mass Directed AutoPurification system.

The ^1^H and ^13^C nucleic magnetic resonance (NMR) spectra were recorded at 400 MHz on a Bruker 400 spectrometer (Bruker Analytische Messtechnik GmbH, Rheinstetten, Germany). Chemical shifts were determined relatively to the signals of residual protons of the deuterated solvent (DMSO). Chemical shifts are reported in delta (δ) units in parts per million (ppm), and splitting patterns are designated as s, singlet; d, doublet; t, triplet; q, quartet; m, multiplet and br, broad. Coupling constants were recorded in Hertz (Hz). High resolution mass spectra were determined on an Agilent 1100 series.

### 4.2. Synthesis

#### 4.2.1. General Procedure for the Preparation of Benzoxaborolic Acids

**Scheme 1:**
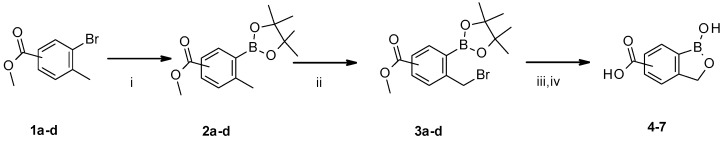
Reagents and conditions: (**i**) Pinacol diborane, PdCl_2_(dppf), KOAc, dioxane, reflux; (**ii**) N-bromosuccinimide (NBS), azobisisobutyronitrile (AIBN), CCl_4_, reflux; (**iii**) Et_2_O, 20% KOH, rt; (**ix**) 0 °C, 6N HCl.

Reactions were performed under argon atmosphere using syringe septa technique. Tolyl bromide **1a–d** (8.731 mmol), pinacol diboron (12.223 mmol, 1.4 eq) and KOAc (26.193 mmol, 3 eq) were dissolved in dioxane (180 mL) at rt, and the resulting mixture was bubbled with argon. PdCl_2_(dppf) (0.873 mol, 0.1 eq) was added and stirring was continued for 16 h at 90 °C. The reaction mixture was cooled to rt, quenched with NH_4_Cl (70 mL), and extracted with Et_2_O (3 × 70 mL). The organic layers were combined, dried over Na_2_SO_4_/MgSO_4_, filtered off end evaporated in vacuo to give raw material which was been purified by Interchim Puriflash 450 in the gradient of MeOH in dichloromethane (DCM): 0%–5% in 20 column volumes. The appropriate fractions were combined and evaporated in vacuo to give corresponding intermediates **2a–d**. [27] To a solution of corresponding intermediates **2a–d** (7.243 mmol) in benzene (40 mL), NBS (7.605 mmol, 1.05 eq) and AIBN (0.362 mmol, 0.05 eq) were added and the reaction mixture stirred at reflux. four hours later solvent had evaporated in vacuo, and Et_2_O was added to the residue (40 mL). The resulting precipitate was filtered off, and the filtrate got extracted with 20% KOH (3 × 30 mL). The aqueous layers were combined and stirred at rt 90 min. The solution was cooled to 0 °C, and pH was adjusted to 2 with 6N aqueous HCl solution. The white precipitate was collected by filtration to afford corresponding benzoxaborole acids **4–7** (Scheme 1).

*methyl 4-methyl-3-(4,4,5,5-tetramethyl-1,3,2-dioxaborolan-2-yl)benzoate 2a* [28]

*3-methyl-4-(4,4,5,5-tetramethyl-1,3,2-dioxaborolan-2-yl)benzoic acid 2b* [29]

*methyl 2-methyl-3-(4,4,5,5-tetramethyl-1,3,2-dioxaborolan-2-yl)benzoate 2c* [22]

*methyl 3-methyl-2-(4,4,5,5-tetramethyl-1,3,2-dioxaborolan-2-yl)benzoate 2d*, Yield: 53%, ^1^H NMR (DMSO*-d6*, 400 MHz, δ ppm): 7.84–7.88 (m, 1H), 7.49–7.57 (m, 2H), 3.97 (s, 3H), 2.49 (s, 3H), 1.47 (s, 12H) ^13^C NMR (DMSO-*d6*, 100 MHz, δ ppm): 167.7, 140.8, 133.6, 132.6, 128.8, 125.7, 83.4, 52.3, 25.0, 21.0 HRMS: m/z calculated for C_15_H_21_BO_4_ [M + H]^+^ 276.1642; found 276.1639.

*1-hydroxy-3H-2,1-benzoxaborole-6-carboxylic acid 4* [30]

*1-hydroxy-3H-2,1-benzoxaborole-5-carboxylic acid 5* [30]

*1-hydroxy-3H-2,1-benzoxaborole-7-carboxylic acid 6* [31]

*1-hydroxy-3H-2,1-benzoxaborole-4-carboxylic acid 7*, Yield: 58%, ^1^H NMR (DMSO*-d6*, 400 MHz, δ ppm): 9.28 (s, 1H), 8.02 (m, 1H), 7.96 (m, 1H), 7.50 (m, 1H), 5.21 (s, 2H) ^13^C NMR (DMSO*-d6*, 100 MHz, δ ppm): 167.2, 134.9, 132.1, 127.4, 71.3 HRMS: m/z calculated for C_8_H_7_BO_4_ [M − H]^−^ 176.0401; found 176.0397.

#### 4.2.2. General Procedure for the Preparation of Carbamate Intermediates

**Scheme 2:**
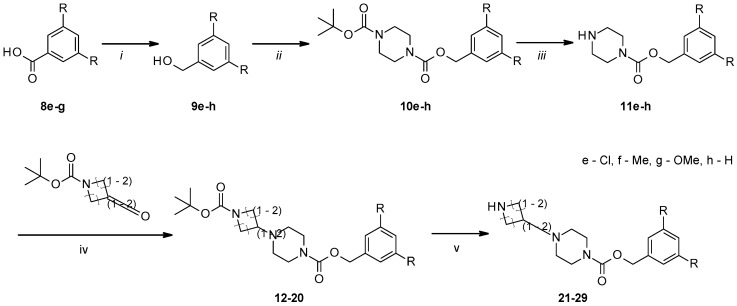
Reagents and conditions: (**i**) LiAlH_4_, THF, 0 °C; (**ii**) *tert*-butyl piperazine-1-carboxylate, CDI, DMF, rt; (**iii**) TFA, dichloromethane (DCM), 0 °C to rt; (**iv**) Boc protected 4-, 5-, or 6-membered ketone, NaBH(OAc)_3_, DCM, 0 °C to rt; (**v**) TFA, DCM, 0 °C to rt.

The first step in the synthetic pathway was the reduction of an appropriate carboxylic acid (**8e–g**) by LiAlH_4_. The entire reaction was performed under an argon atmosphere using a syringe septa technique. To a solution of lithium aluminum hydride (0.063 mol, 1.2 eq) in THF (15 mL) that was cooled in an ice bath, a solution of the substituted benzoic acid (**8e–g**) (0.052 mol, 1 eq) in THF (65 mL) was added dropwise during 30 minutes. The resulting mixture was stirred at ambient temperature for 2 h. To the reaction mixture were added: Water (3 mL), a 1M solution of NaOH (3 mL), and then water (10 mL) whilst stirring continued for a further 90 minutes at ambient temperature. The mixture was extracted with EtOAc (3 × 30 mL). The organic layers were combined, dried over Na_2_SO_4_/MgSO_4_, filtered and evaporated in vacuo to give a crude product which was purified by Interchim Puriflash 450 with a gradient of EtOAc in cyclohexane. The appropriate fractions were combined and evaporated in vacuo to give corresponding alcohols (**9e–g**). The resultant alcohols (0.039 mol, 1 eq) and CDI (0.039 mol, 1 eq) were dissolved in DMF (100 mL) at ambient temperature, and the resulting mixture was treated with the Boc protected piperazine (0.035 mol, 0.9 eq), and stirring was continued for 16 h at ambient temperature. Solvent was evaporated in vacuo. Water (50 mL) was added to the residue. Extraction with dichloromethane (DCM) (3 × 30 mL) followed. The organic layers were combined, dried over Na_2_SO_4_/MgSO_4_, filtered off, end evaporated in vacuo to give raw material which was purified by Interchim Puriflash 450 in the gradient of MeOH in DCM: 0%–3% in 20 CV. The appropriate fractions were combined and evaporated in vacuo to give corresponding Boc protected piperazine carbamates (**10e–h**). To a solution of Boc protected piperazine carbamate (**10e–h**) (0.035 mol, 1 eq) in DCM (200 mL), cooled in an ice bath, TFA (0.353 mol, 10 eq) was added. The resulting mixture was stirred at ambient temperature for 30 minutes. The pH of the reaction mixture was adjusted to 8 with 6N aqueous solution of NaOH and layers were separated. The aqueous layer was washed with DCM (2 × 100 mL). The organic layers were combined, dried over Na_2_SO_4_/MgSO_4_, filtered off and evaporated in vacuo to give corresponding piperazine carbamates intermediates (**11e–h**). The piperazine carbamate intermediates (**11e–h**) (0.010 mol, 1 eq) and corresponding N-Boc protected cyclic ketone (0.010 mol, 1 eq) were dissolved in DCM (45 mL) at rt, and to the resulting solution NaBH(OAc)_3_ (0.013 mol, 1.3 eq) was added in portions before stirring was continued for 16 h at rt. The reaction mixture was quenched with a saturated solution of NaHCO_3_ (30 mL). The layers were separated, and the aqueous layer was washed with DCM (2 × 20 mL). The organic layers were combined, dried over Na_2_SO_4_/MgSO_4_, filtered off and evaporated in vacuo to give crude product which was purified by Interchim Puriflash 450, in a gradient of MeOH in DCM: 0%–5% in 20 column volumes. The appropriate fractions were combined and evaporated in vacuo to give corresponding Boc protected intermediates (**12–20**). Finally, the Boc protecting group was cleaved with TFA to obtain carbamate intermediates (**21–29**) (Scheme 2).

*O4-tert-butyl O1-[(3,5-dichlorophenyl)methyl] piperazine-1,4-dicarboxylate* [32]

O4-tert-butyl O1-[(3,5-dimethylphenyl)methyl] piperazine-1,4-dicarboxylate 10f, Yield: 86%, ^1^H NMR (DMSO*-d6*, 400 MHz, δ ppm): 6.94 (s, 3H), 4.99 (s, 2H), 3.32–3.39 (m, 2H), 2.25 (s, 6H), 1.39 (s, 9H) ^13^C NMR (DMSO*-d6*, 100 MHz, δ ppm): 179.3, 154.0, 153.7, 137.4, 136.5, 129.5, 125.3, 79.1, 66.4, 43.2, 28.0, 20.8 HRMS: m/z calculated for C_15_H_21_BO_4_ [M + H-100]^+^ 276.1642; found 276.1641.

O4-tert-butyl O1-[(3,5-dimethoxyphenyl)methyl] piperazine-1,4-dicarboxylate 10g, Yield: 83%, ^1^H NMR (DMSO-*d6*, 400 MHz, δ ppm): 7.84–7.88 (m, 2H), 7.49–7.57 (m, 1H), 5.02 (s, 2H), 3.72 (s, 6H), 3.32–3.39 (m, 2H), 1.47 (s, 12H) ^13^C NMR (DMSO*-d6*, 100 MHz, δ ppm): 179.7, 179.5, 154.3, 153.7, 139.0, 105.3, 99.4, 79.1, 66.1, 55.1, 27.9 HRMS: m/z calculated for C_19_H_28_N_2_O_6_ [M + Na]^+^ 403.1840; found 403.1825.

*O1-benzyl O4-tert-butyl piperazine-1,4-dicarboxylate 10h* [33]

*(3,5-dichlorophenyl)methyl piperazine-1-carboxylate11e* [34]

(3,5-dimethylphenyl)methyl piperazine-1-carboxylate 11f, Yield: 98%, ^1^H NMR (DMSO-*d6*, 400 MHz, δ ppm): 8.19 (br. s, 1H), 6.93 (m, 3H), 5.00 (s, 2H), 2.62–2.70 (m, 2H), 2.25 (s, 6H) ^13^C NMR (DMSO*-d6*, 100 MHz, δ ppm): 179.7, 179.5, 163.2, 161.0, 137.4, 129.3, 129.2, 125.5, 125.4, 70.0, 66.5, 66.2, 45.1, 20.8 HRMS: m/z calculated for C_14_H_20_N_2_O_2_ [M + H]^+^ 249.1598; found 249.1599.

(3,5-dimethoxyphenyl)methyl piperazine-1-carboxylate 11g, Yield: 97%, ^1^H NMR (DMSO*-d6*, 400 MHz, δ ppm): 6.39–6.45 (m, 3H), 4.99 (s, 2H), 3.72 (s, 6H), 2.58–2.68 (m, 2H) ^13^C NMR (DMSO*-d6*, 100 MHz, δ ppm): 179.2, 160.5, 160.4, 154.4, 139.3, 105.3, 105.1, 99.3, 65.8, 55.2, 55.1, 45.3, 44.5 HRMS: m/z calculated for C_14_H_20_N_2_O_4_ [M + H]^+^ 281.1496; found 281.1522.

*benzyl piperazine-1-carboxylate11h* [35]

*(3,5-dichlorophenyl)methyl 4-(1-tert-butoxycarbonylazetidin-3-yl)piperazine-1-carboxylate 12*, Yield: 72%, ^1^H NMR (DMSO*-d6*, 400 MHz, δ ppm): 7.74–7.85 (m, 1H), 7.5–7.72 (m, 2H), 5.30 (s, 2H), 3.99–4.12 (m., 2H), 3.80–3.94 (m, 2H), 3.52–3.71 (m, 4H), 1.60 (s, 9H) ^13^C NMR (DMSO*-d6*, 100 MHz, δ ppm): 155.6, 154.1, 141.2, 134.1, 127.7, 126.1, 78.6, 64.7, 53.0, 48.6, 43.2, 28.0 HRMS: m/z calculated for C_20_H_27_Cl_2_N_3_O_4_ [M + H]^+^ 444.1451; found 444.1450.

*(3,5-dichlorophenyl)methyl 4-(1-tert-butoxycarbonylpyrrolidin-3-yl)piperazine-1-carboxylate 13,* Yield: 60%, ^1^H NMR (DMSO-*d6*, 400 MHz, δ ppm) 7.52–7.60 (m, 1H), 7.36–7.47 (m, 2H), 5.03 (s, 2H), 3.37–3.56 (m, 4H), 3.09–3.21 (m, 1H), 3.00–3.07 (m, 1H), 2.84–2.99 (m, 1H), 2.64–2.87 (m, 1H), 2.37–2.45 (m, 1H), 1.89–1.92 (m., 1H), 1.37 (s, 9H) ^13^C NMR (DMSO*-d6*, 100 MHz, δ ppm): 153.9, 141.2, 134.1, 127.4, 126.3, 126.2, 115.3, 64.8, 64.7, 64.4, 64.7, 50.8, 49.2, 44.7, 28.9 HRMS: m/z calculated for C_21_H_29_Cl_2_N_3_O_4_ [M + H]^+^ 458.1608; found 458.1625.

*(3,5-dichlorophenyl)methyl 4-(1-tert-butoxycarbonyl-4-piperidyl)piperazine-1-carboxylate 14*, Yield: 77%, ^1^H NMR (DMSO-*d6*, 400 MHz δ ppm) 7.60 (s, 1H), 7.41–7.51 (m, 2H), 5.11 (s, 2H), 3.92–4.04 (m, 2H), 3.39–3.49 (m, 2H), 2.69–2.76 (m, 2H), 2.38–2.52 (m, 2H), 1.69–1.71 (m, 2H), 1.43 (s, 9H), 1.20–1.35 (m, 2H) ^13^C NMR (DMSO*-d6*, 100 MHz, δ ppm): 154.0, 153.8, 141.3, 134.0, 127.4, 126.1, 78.5, 64.6, 60.8, 48.3, 43.9, 28.0, 27.6 HRMS: m/z calculated for C_22_H_31_Cl_2_N_3_O_4_ [M + H]^+^ 472.1764; found 472.1774.

*(3,5-dimethylphenyl)methyl 4-(1-tert-butoxycarbonylazetidin-3-yl)piperazine-1-carboxylate 15*, Yield: 67%, ^1^H NMR (DMSO-*d6*, 400 MHz δ ppm) 7.89–7.96 (m, 3H), 4.97 (s, 2H), 3.75–3.83 (m, 2H), 3.59–3.68 (m, 2H), 3.35–3.39 (m, 2H), 2.97–3.06 (m,1H), 2.24 (s, 6H), 2.17–2.23 (m, 2H), 1.47 (s, 9H) ^13^C NMR (DMSO*-d6*, 100 MHz, δ ppm): 179.4, 155.6, 154.4, 137.4, 136.6, 129.2, 125.5, 125.4, 78.6, 66.3, 53.0, 48.7, 43.2, 28.0, 20.8 HRMS: m/z calculated for C_22_H_33_N_3_O_4_ [M + H]^+^ 404.2544; found 404.2561.

*(3,5-dimethylphenyl)methyl 4-(1-tert-butoxycarbonylpyrrolidin-3-yl)piperazine-1-carboxylate 16*, Yield: 59%, ^1^H NMR (DMSO-*d6*, 400 MHz, δ ppm) 7.01 (s, 3H), 5.08 (s, 2H), 3.38–3.44 (m, 4H), 3.11–3.26 (m, 1H), 2.91–3.03 (m, 1H), 2.71–2.87 (m, 1H), 2.39–2.49 (m, 1H), 2.30–2.39 (m, 1H), 2.29 (s, 6H), 1.61–1.75 (m, 1H), 1.42 (s, 9H) ^13^C NMR (DMSO*-d6,* 100 MHz, δ ppm): 179.0, 154.3, 153.4, 137.4, 136.6, 129.2, 125.4, 78.3, 66.2, 63.5, 62.7, 50.8, 49.3, 49.2, 44.6, 44.4, 43.3, 28.9, 28.1, 20.8 HRMS: m/z calculated for C_23_H_36_N_3_O_4_ [M + H]^+^ 418.2711; found 418.2718.

*(3,5-dimethoxyphenyl)methyl 4-(1-tert-butoxycarbonylazetidin-3-yl)piperazine-1-carboxylate 17*, Yield: 74%, ^1^H NMR (DMSO-*d6*, 400 MHz, δ ppm) 6.45–6.51 (m, 2H), 6.42 (s, 1H), 4.99 (s, 2H), 3.75–3.87 (m, 2H), 3.72 (s, 6H), 3.59–3.68 (m, 2H), 3.34–3.46 (m, 4H), 2.98–3.07 (m, 1H), 1.36 (s, 9H) ^13^C NMR (DMSO*-d6*, 100 MHz, δ ppm): 179.1, 160.5, 155.5, 154.3, 139.2, 105.2, 99.3, 78.6, 66.0, 55.2, 55.1, 53.0, 48.7, 43.2, 28.0 HRMS: m/z calculated for C_22_H_33_N_3_O_6_ [M + H]^+^ 436.2442; found 436.2428.

*(3,5-dimethoxyphenyl)methyl 4-(1-tert-butoxycarbonylpyrrolidin-3-yl)piperazine-1-carboxylate 18*, Yield: 55%, ^1^H NMR (DMSO-*d6*, 400 MHz, δ ppm) 6.46–6.49 (m, 2H), 6.40–6.45 (m, 1H), 4.99 (s, 2H), 3.72 (s, 6H), 3.45–3.53 (m, 1H), 3.35–3.42 (m, 4H), 2.87–2.98 (m, 1H), 2.61–2.69 (m, 1H), 2.34–2.43 (m, 1H), 2.27–2.35 (m, 1H), 1.94–2.06 (m, 1H), 1.37 (s, 9H) ^13^C NMR (DMSO*-d6*, 100 MHz, δ ppm): 179.5, 160.5, 105.2, 99.3, 65.9, 55.2 28.1 HRMS: m/z calculated for C_22_H_35_N_3_O_6_ [M + H]^+^ 450.2599 found 450.2641.

*benzyl 4-(1-tert-butoxycarbonylazetidin-3-yl)piperazine-1-carboxylate19* [36]

*benzyl 4-(1-tert-butoxycarbonylpyrrolidin-3-yl)piperazine-1-carboxylate20* [37]

*(3,5-dichlorophenyl)methyl 4-(azetidin-3-yl)piperazine-1-carboxylate 21*, Yield: 72%, ^1^H NMR (DMSO*-d6*, 400 MHz, δ ppm): 7.51–7.62 (m, 1H), 7.38–7.42 (m, 2H), 5.06 (s, 2H), 4.07–4.16 (m., 2H), 3.78–3.14 (m, 2H), 3.52–3.71 (m, 2H), ^13^C NMR (DMSO-*d6*, 100 MHz, δ ppm): 179.1, 161.1, 154.1, 141.2, 134.1, 127.5, 126.2, 64.8, 54.8, 53.0, 50.5, 48.6, 43.2 HRMS: m/z calculated for C_15_H_19_Cl_2_N_3_O_2_ [M + H]^+^ 344.0922; found 344.0931.

*3,5-dichlorophenyl)methyl 4-pyrrolidin-3-ylpiperazine-1-carboxylate 22*, Yield: 99%, ^1^H NMR (DMSO-*d6*, 400 MHz, δ ppm) 7.52–7.60 (m, 1H), 7.36–7.47 (m, 2H), 5.03 (s, 2H), 3.00–3.07 (m, 1H), 2.84–2.99 (m, 1H), 2.75–2.83 (m, 1H), 2.64–2.87 (m, 1H), 2.59–2.64 (m, 1H), 1.87–1.89 (m, 1H), 1.55–1.68 (m, 1H) ^13^C NMR (DMSO-*d6,* 100 MHz, δ ppm): 154.0, 141.2, 134.1, 127.4, 126.2, 126.1, 64.7, 64.1, 51.0, 48.6, 44.7, 43.4, 28.5 HRMS: m/z calculated for C_16_H_21_Cl_2_N_3_O_2_ [M + H]^+^ 458.1608; found 458.1625.

*(3,5-dichlorophenyl)methyl 4-(4-piperidyl)piperazine-1-carboxylate 23*, Yield: 99%, ^1^H NMR (DMSO-*d6*, 400 MHz, δ ppm) 7.52–7.60 (m, 1H), 7.36–7.47 (m, 2H), 5.03 (s, 2H), 3.92–4.04 (m, 2H), 3.39–3.49 (m, 2H), 2.69–2.76 (m, 2H), 2.38–2.52 (m, 2H), 1.69–1.71 (m, 2H), 1.20–1.35 (m, 2H) ^13^C NMR (DMSO-d6, 100 MHz, δ ppm): 154.0, 153.8, 141.3, 134.0, 127.4, 126.1, 64.6, 48.3, 43.9 HRMS: m/z calculated for C_17_H_23_Cl_2_N_3_O_2_ [M + H]^+^ 372.124; found 372.1237.

*(3,5-dimethylphenyl)methyl 4-(azetidin-3-yl)piperazine-1-carboxylate 24*, Yield: 99%, ^1^H NMR (DMSO-*d6*, 400 MHz, δ ppm) 6.94–7.96 (m, 3H), 4.99 (s, 2H), 3.72–3.79 (m, 2H), 3.64–3.71 (m, 2H), 2.25 (s, 6H) ^13^C NMR (DMSO-*d6*, 100 MHz, δ ppm): 179.5, 165.4, 154.4, 137.4, 136.6, 129.3, 125.4, 66.3, 56.0, 54.7, 49.4, 48.4, 20.9, 20.8 HRMS: m/z calculated for C_22_H_33_N_3_O_4_ [M + H]^+^ 404.2544; found 404.2561.

*(3,5-dimethylphenyl)methyl 4-pyrrolidin-3-ylpiperazine-1-carboxylate 25*, Yield: 99%, ^1^H NMR (DMSO-*d6*, 400 MHz, δ ppm) 6.89–7.05 (m, 3H), 5.01 (s, 2H), 3.73 (m, 1H), 3.31–3.46 (m, 1H), 3.11–3.26 (m, 1H), 2.91–3.03 (m, 1H), 2.71–2.87 (m, 1H), 2.39–2.49 (m, 1H), 2.30–2.39 (m, 1H), 2.29 (s, 6H), 1.61–1.75 (m, 1H) ^13^C NMR (DMSO*-d6*, 100 MHz, δ ppm): 179.5, 154.4, 137.4, 136.6, 129.3, 125.4, 66.3, 62.8, 50.7, 47.2, 44.0, 43.3, 27.4, 20.9 HRMS: m/z calculated for C_18_H_27_N_3_O_2_ [M + H]^+^ 318.2181; found 318.2186.

*(3,5-dimethoxyphenyl)methyl 4-(azetidin-3-yl)piperazine-1-carboxylate 26*, Yield: 95%, ^1^H NMR (DMSO-*d6*, 400 MHz, δ ppm) 6.45–6.51 (m, 2H), 6.42 (s, 1H), 4.99 (s, 2H), 3.75–3.87 (m, 2H), 3.72 (s, 6H), 3.59–3.68 (m, 2H), 3.34–3.46 (m, 2H), 2.98–3.07 (m, 1H), 2.24 (m, 4H) ^13^C NMR (DMSO-*d6*, 100 MHz, δ ppm): 179.6, 160.5, 154.3, 139.2, 105.2, 99.3, 66.0, 56.0, 55.2, 49.2, 48.4, 43.1 HRMS: m/z calculated for C_17_H_25_N_3_O_4_ [M + H]^+^ 336.1918; found 336.1939.

*(3,5-dimethoxycyclohexa-1,5-dien-1-yl)methyl 4-pyrrolidin-3-ylpiperazine-1-carboxylate 27*, Yield: 93%, ^1^H NMR (DMSO-*d6*, 400 MHz, δ ppm) 6.46–6.49 (m, 2H), 6.40–6.45 (m, 1H), 4.99 (s, 2H), 3.72 (s, 6H), 3.00–3.18 (m, 2H), 2.87–2.98 (m, 1H), 2.70–2.83 (m, 1H), 2.59–2.63 (m, 1H), 2.37–2.47 (m, 2H), 1.87–1.96 (m, 1H), 1.54–1.67 (m, 1H) ^13^C NMR (DMSO*-d6,* 100 MHz, δ ppm): 179.5, 160.5, 154.2, 139.2, 150.2, 105.1, 99.3, 66.0, 64.3, 55.2, 41.1, 48.3, 28.5 HRMS: m/z calculated for C_18_H_27_N_3_O_4_ [M + H]^+^ 350.2074; found 350.2089.

*benzyl 4-(azetidin-3-yl)piperazine-1-carboxylate28* [28]

*benzyl 4-pyrrolidin-3-ylpiperazine-1-carboxylate29* [28]

#### 4.2.3. General Procedure for the Preparation of Novel Autotaxin Inhibitors

**Scheme 3:**
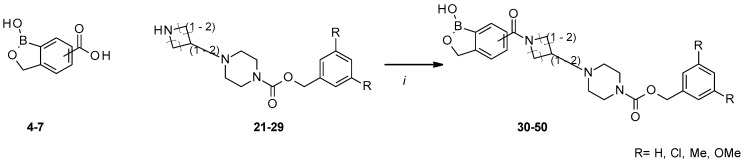
Reagents and conditions: (i) N-(3-Dimethylaminopropyl)-N′-ethylcarbodiimide (EDAC), 4-dimethylaminopyridine (DMAP), DCM, rt.

To a solution of benzoxaborolic acid (**4–7**) (0.232 mmol, 1 eq), the carboxylate intermediate (**21–29**) (0.232 mmol) in DCM (1 mL), EDAC (0.372 mmol, 1.6 eq) and DMAP (0.009 mmol, 0.04 eq) were added at ambient temperature, and the resulting mixture was stirred for 16 h. The solvent was evaporated in vacuo, and the raw material was purified by a Waters Mass Directed AutoPurification system, giving corresponding novel autotaxin inhibitors (**30–50**) (Scheme 3).

*(3,5-dichlorophenyl)methyl 4-[1-(1-hydroxy-3H-2,1-benzoxaborole-6-carbonyl)pyrrolidin-3-yl]piperazine-1-carboxylate30*, Yield: 79%, ^1^H NMR (DMSO-d6, 400 MHz, δ ppm): 9.29 (br.s, 1H), 7.87 (d, *J* = 7.6 Hz, 1H), 7.61 (d, *J* = 7.5 Hz, 1H), 7.55 (s, 1H), 7.35–7.48 (m, 3H), 5.05 (s, 2H), 5.01 (s, 2H), 3.71–3.80 (m, 2H), 3.58–3.67 (m, 2H), 3.50–3.58 (m, 2H), 2.84–2.94 (m, 2H), 2.73–2.84 (m, 2H), 2.34–2.47 (m, 2H), 2.08–2.18 (m, 2H), ^13^C NMR (DMSO-d6, 400 MHz, δ ppm): 168.6, 141.2, 134.1, 129.7, 129.3, 127.5, 126.2, 121.3, 69.9, 67.9, 63.7, 62.4, 54.3, 25.9 HREI-MS: m/z calculated for C_24_H_26_BCl_2_N_3_O_5_ [M]^+^ 517.1452; found 517.1448.

*(3,5-dichlorophenyl)methyl 4-[1-(1-hydroxy-3H-2,1-benzoxaborole-7-carbonyl)pyrrolidin-3-yl]piperazine-1-carboxylate31*, Yield: 53%, ^1^H NMR (DMSO-d6, 400 MHz, δ ppm): 9.28 (br.s, 1H), 7.78 (d, *J* = 7.7 Hz, 1H), 7.69–7.73 (m, 1H),7.56 (s, 1H) 7.48–7.51 (m, 1H), 7.39–7.43 (m, 1H), 5.05 (s, 2H), 5.01 (s, 2H), 3.70–3.82 (m, 2H), 3.52–3.63 (m, 2H), 3.26–3.42 (m, 4H), 3.18–3.29 (m, 1H), 2.34–2.43 (m, 4H), 1.99–2.15 (m, 2H), ^13^C NMR (DMSO-d6, 400 MHz, δ ppm): 168.6, 141.2, 134.1, 129.7, 129.3, 127.5, 126.2, 121.3, 69.9, 67.9, 63.7, 62.4, 54.3, 25.9, HREI-MS: m/z calculated for C_24_H_26_BCl_2_N_3_O_5_ [M]^+^ 517.1452; found 517.1446.

*(3,5-dichlorophenyl)methyl 4-[1-(1-hydroxy-3H-2,1-benzoxaborole-5-carbonyl)pyrrolidin-3-yl]piperazine-1-carboxylate 32*, Yield: 68%, ^1^H NMR (DMSO-d6, 400 MHz, δ ppm): 9.33 (br.s, 1H), 7.66–7.88 (m, 1H), 7.47–7.59 (m, 2H), 7.35–7.46 (m, 3H), 5.04 (s, 2H), 5.00 (s, 2H), 3.70–3.76 (m, 2H), 3.48–3.62 (m, 2H), 2.85–2.92 (m, 4H), 2.06–2.17 (m, 4H), 1.95–2.06 (m, 2H) ^13^C NMR (DMSO-d6, 400 MHz, δ ppm): 168.4, 134.1, 131.9, 129.9, 127.4, 126.2, 125.8, 120.0, 99.8, 98.6, 62.0, 25.2, 19.7, 8.1, HREI-MS: m/z calculated for C_24_H_26_BCl_2_N_3_O_5_ [M]^+^ 517.1452; found 517.1451.

*(3,5-dichlorophenyl)methyl 4-[1-(1-hydroxy-3H-2,1-benzoxaborole-4-carbonyl)pyrrolidin-3-yl]piperazine-1-carboxylate) 33*, Yield: 59%, ^1^H NMR (DMSO-d6, 400 MHz, δ ppm): 9.32 (br.s, 1H), 7.82–7.86 (m, 1H), 7.71–7.74 (m, 1H), 7.62–7.58 (m, 1H), 7.34–7.42 (m, 2H),7.41–7.51 (m, 1H), 5.03 (s, 2H), 5.00 (s, 2H), 3.70–3.82 (m, 2H), 3.37–3.54 (m, 2H), 3.23–3.32 (m, 5H), 2.15–2.25 (m, 4H), 1.96–2.25 (m, 2H), ^13^C NMR (DMSO-d6, 400 MHz, δ ppm): 168.4, 134.1, 131.9, 129.9, 127.4, 126.2, 125.8, 120.0, 99.8, 98.6, 62.0, 25.2, 19.7, 8.1 HREI-MS: m/z calculated for C_24_H_26_BCl_2_N_3_O_5_ [M]^+^ 517.1452; found 517.1448.

*3,5-dichlorophenyl)methyl 4-[1-(1-hydroxy-3H-2,1-benzoxaborole-6-carbonyl)-4-piperidyl]piperazine-1-carboxylate34*, Yield: 81%, ^1^H NMR (DMSO-d6, 400 MHz, δ ppm): 9.31 (br.s., 1H), 7.71 (d, *J* = 7.6 Hz, 1H), 7.54 (s, 1H), 7.45–7.47 (m, 2H) 7.38–7.42 (m, 2H), 5.05 (s, 2H), 5.00 (s, 2H), 4.41–4.53 (m, 1H), 3.54–3.65 (m, 1H), 2.93–3.07 (m, 1H), 2.70–2.82 (m, 1H), 2.50–2.54 (m, 1H), 2.44–2.54 (m, 4H), 2.43–2.47 (m, 4H), 1.77–1.87 (m, 1H), 1.63–1.74 (m, 1H), 1.29–1.42 (m, 2H) ^13^C NMR (DMSO-d6, 400 MHz, δ ppm): 169.1, 154.9, 154.0, 141.2, 134.9, 134.0, 129.2, 128.7, 127.4, 126.1, 121.4, 69.9, 64.7, 60.8, 48.4, 43.9, HREI-MS: m/z calculated for C_25_H_28_BCl_2_N_3_O_5_ [M]^+^ 531.1608; found 531.1606.

*(3,5-dichlorophenyl)methyl 4-[1-(1-hydroxy-3H-2,1-benzoxaborole-5-carbonyl)-4-piperidyl]piperazine-1-carboxylate35*, Yield: 73%, ^1^H NMR (DMSO-d6, 400 MHz, δ ppm): 9.32 (br.s., 1H), 7.75 (d, *J =* 7.6 Hz, 1H), 7.54 (s, 1H), 7.40 (d, *J =* 7.4 Hz, 1H), 7.5–7.42 (m, 2H), 7.29–7.34 (m, 1H), 5.05 (s, 2H), 4.99 (s, 2H), 4.20–4.42 (m, 1H), 3.50–3.60 (m, 2H), 2.94–3.05 (m, 2H), 2.70–2.80 (m, 1H), 2.50–2.54 (m, 1H), 2.44–2.54 (m, 4H), 2.43–2.47 (m, 4H), 1.77–1.86 (m, 1H), 1.60–1.69 (m, 1H) ^13^C NMR (DMSO-d6, 400 MHz, δ ppm): 168.8, 154.0, 141.5, 138.4, 134.0, 130.5, 127.3, 126.3, 125.1, 119.5, 69.9, 64.8, 60.7, 48.3, 43.9, 28.4, HREI-MS: m/z calculated for C_25_H_28_BCl_2_N_3_O_5_ [M]^+^ 531.1608; found 531.1607.

*(3,5-dichlorophenyl)methyl 4-[1-(1-hydroxy-3H-2,1-benzoxaborole-4-carbonyl)-4-piperidyl]piperazine-1-carboxylate 36*, Yield: 59%, ^1^H NMR (DMSO-d6, 400 MHz, δ ppm): 9.93 (s, 1H), 7.52–7.62 (m, 2H), 7.41 (s, 1H), 7.20–7.34 (m, 2H), 7.15–7.18 (m, 1H), 5.06 (s, 4H), 4.30–4.41 (m, 1H), 3.87–4.00 (m, 2H), 2.91–3.05 (m, 2H), 2.67–2.80 (m, 1H), 2.57–2.66 (m, 4H), 2.50–2.54 (m, 1H), 1.62–1.83 (m, 1H) ^13^C NMR (DMSO-d6, 400 MHz, δ ppm): 168.8, 154.0, 141.5, 138.4, 134.0, 130.5, 127.3, 126.3, 125.1, 119.5, 69.9, 64.8, 60.7, 48.3, 43.9, 28.4 HREI-MS: m/z calculated for C_25_H_28_BCl_2_N_3_O_5_ [M]^+^ 531.1608; found 531.1605.

*(3,5-dichlorophenyl)methyl 4-[1-(1-hydroxy-3H-2,1-benzoxaborole-6-carbonyl)azetidin-3-yl]piperazine-1-carboxylate 37*, Yield: 83%, ^1^H NMR (DMSO-d6, 400 MHz, δ ppm): 9.29 (br.s., 1H), 8.00–8.02 (m, 1H), 7.73 (d, *J =* 7.7 Hz, 1H), 7.55 (s, 1H), 7.47 (d, *J =* 7.9 Hz, 1H), 7.40 (d, *J =* 1.9 Hz, 2H), 5.06 (s, 2H), 5.02 (s, 2H), 4.03–4.14 (m, 2H), 3.84–3.90 (m, 1H), 3.36–3.48 (m, 4H), 3.13–3.21 (m, 1H), 2.25–2.36 (m, 4H), ^13^C NMR (DMSO-d6, 400 MHz, δ ppm): 169.2, 156.7, 154.0, 141.3, 134.0, 131.7, 129.9, 127.5, 126.2, 121.6, 70.2, 64.8, 53.9, 48.9, 43.2, HREI-MS: m/z calculated for C_23_H_24_BCl_2_N_3_O_5_ [M]^+^ 503.1295; found 503.1299.

*(3,5-dichlorophenyl)methyl 4-[1-(1-hydroxy-3H-2,1-benzoxaborole-5-carbonyl)azetidin-3-yl]piperazine-1-carboxylate 38*, Yield: 77%, ^1^H NMR (DMSO-d6, 400 MHz, δ ppm): 9.33 (br.s., 1H), 7.78 (d, *J =* 7.8 Hz, 1H), 7.62–7.64 (m, 1H), 7.54–7.58 (m, 2H), 7.41 (d, *J =* 1.9 Hz, 2H), 5.06 (s, 2H), 5.00 (s, 2H), 4.20–4.28 (m,2H), 3.99–4.12 (m, 2H), 3.09–3.20 (m, 1H), 2.24–2.32 (m, 4H), ^13^C NMR (DMSO-d6, 400 MHz, δ ppm): 169.2, 156.7, 154.0, 141.3, 134.0, 131.7, 129.9, 127.5, 126.2, 121.6, 70.2, 64.8, 53.9, 48.9, 43.2, HREI-MS: m/z calculated for C_23_H_24_BCl_2_N_3_O_5_ [M]^+^ 503.1295; found 503.1301.

*benzyl 4-[1-(1-hydroxy-3H-2,1-benzoxaborole-6-carbonyl)pyrrolidin-3-yl]piperazine-1-carboxylate 39*, Yield: 82%, ^1^H NMR (DMSO-d6, 400 MHz, δ ppm): 9.26 (br.s, 1H), 7.70–7.80 (m, 1H), 7.54–7.70 (m, 1H), 7.41–7.49 (m, 1H), 7.24–7.41 (m, 5H), 5.06 (s, 2H), 5.01 (s, 2H), 3.71–3.79 (m, 2H), 3.58–3.67 (m, 2H), 3.49–3.57 (m, 2H), 2.84–2.92 (m, 2H), 2.74–2.83 (m, 2H), 2.34–2.43 (m, 2H), 2.07–2.17 (m, 2H), ^13^C NMR (DMSO-d6, 400 MHz, δ ppm): 168.5, 155.5, 154.2, 136.8, 135.4, 129.6, 129.2, 128.4, 127.9, 121.3, 69.9, 66.2, 63.7, 62.2, 52.5, 50.7, 47.9, 43.3, HREI-MS: m/z calculated for C_24_H_28_BN_3_O_5_ [M]^+^ 449.2231; found 449.2240.

*benzyl 4-[1-(1-hydroxy-3H-2,1-benzoxaborole-5-carbonyl)pyrrolidin-3-yl]piperazine-1-carboxylate 40*, Yield: 71%, ^1^H NMR (DMSO-d6, 400 MHz, δ ppm): 9.30 (br.s, 1H), 7.71–7.81 (m, 1H), 7.48–7.53 (m, 1H), 7.40–7.47 (m, 1H), 7.27–7.38 (m, 5H), 5.05 (s, 2H), 5.00 (s, 2H), 3.72–3.79 (m, 2H), 3.58–3.67 (m, 2H), 3.45–3.53 (m, 4H), 3.38–3.44 (m, 4H), 3.19–3.29 (m, 1H), 2.06–2.28 (m, 2H), ^13^C NMR (DMSO-d6, 400 MHz, δ ppm): 168.4, 154.5, 154.0, 138.6, 137.0, 132.0, 128.5, 127.8, 127.5, 125.6, 120.0, 69.9, 66.1, 63.6, 62.3, 50.7, 43.4, HREI-MS: m/z calculated for C_24_H_28_BN_3_O_5_ [M]^+^ 449.2231; found 449.2243.

*benzyl 4-[1-(1-hydroxy-3H-2,1-benzoxaborole-6-carbonyl)azetidin-3-yl]piperazine-1-carboxylate 41,* Yield: 87%, ^1^H NMR (DMSO-d6, 400 MHz, δ ppm): 9.31 (br.s., 1H), 7.78 (d, *J = 7*.9 Hz, 1H), 7.65 (s, 1H), 7.58 (d, *J =* 7.7 Hz, 1H), 7.29–7.39 (m, 5H), 5.07 (s, 2H), 5.03 (s, 2H), 4.03–4.15 (m, 2H), 3.85–3.91 (m, 1H), 3.39–3.46 (m, 4H), 3.14–3.21 (m, 1H), 2.25–2.34 (m, 4H), ^13^C NMR (DMSO-d6, 400 MHz, δ ppm): 169.5, 156.5, 154.5, 137.1 132.0, 130.2, 128.5, 127.8, 127.6, 121.5, 69.9, 66.1, 57.1, 53.5, 52.9, 48.7, 43.1, HREI-MS: m/z calculated for C_23_H_26_BN_3_O_5_ [M]^+^ 435.2075; found 435.2079.

*benzyl 4-[1-(1-hydroxy-3H-2,1-benzoxaborole-5-carbonyl)azetidin-3-yl]piperazine-1-carboxylate 42*, Yield: 53%, ^1^H NMR (DMSO-d6, 400 MHz, δ ppm): 9.33 (br.s., 1H), 7.78 (d, *J =* 7.6 Hz, 1H), 7.65 (s, 1H), 7.58 (d, *J =* 7.6 Hz, 1H), 7.29–7.39 (m, 5H), 5.07 (s, 2H), 5.03 (s, 2H), 4.04–4.14 (m, 2H), 3.85–3.90 (m, 2H), 3.37–3.45 (m, 4H), 3.12–3.19 (m, 1H), 2.24–2.35 (m, 4H), ^13^C NMR (DMSO-d6, 400 MHz, δ ppm): 169.1, 154.5, 153.9, 136.8, 135.0, 134.4, 130.5, 128.5, 127.9, 126.4, 120.4, 69.9, 66.2, 56.5, 53.6, 52.2, 48.7, 43.1, HREI-MS: m/z calculated for C_23_H_26_BN_3_O_5_ [M]^+^ 435.2075; found 435.2078.

*(3,5-dimethylphenyl)methyl 4-[1-(1-hydroxy-3H-2,1-benzoxaborole-6-carbonyl)pyrrolidin-3-yl]piperazine-1-carboxylate 43*, Yield: 79%, ^1^H NMR (DMSO-d6, 400 MHz, δ ppm): 9.32 (br.s, 1H), 7.79–7.92 (m, 1H), 7.57–7.68 (m, 1H), 7.45 (d, *J* = 7.8 Hz, 1H), 6.91–7.02 (m, 3H), 5.01 (s, 2H), 4.97 (s, 2H), 3.69–3.82 (m, 2H), 3.54–3.67 (m, 4H), 3.32–3.51 (m, 2H), 2.17–2.38 (m, 1H), 1.97–2.06 (m, 1H), ^13^C NMR (DMSO-d6, 400 MHz, δ ppm): 168.5, 155.4, 137.5, 136.6, 129.6, 129.3, 125.5, 121.3, 63.8, 62.3, HREI-MS: m/z calculated for C_26_H_32_BN_3_O_5_ [M]^+^ 477.2544; found 477.2546.

*(3,5-dimethylphenyl)methyl 4-[1-(1-hydroxy-3H-2,1-benzoxaborole-5-carbonyl)pyrrolidin-3-yl]piperazine-1-carboxylate 44*, Yield: 52%, ^1^H NMR (DMSO-d6, 400 MHz, δ ppm): 9.34 (br.s, 1H), 7.76–(d, *J =* 7.5 Hz, 1H), 7.56 (s, 1H), 7.47–7.55 (m, 1H), 6.88–6.97 (m, 3H), 4.92–5.03 (m, 4H), 3.70–3.82 (m, 2H), 3.37–3.45 (m, 2H), 3.23–3.32 (m, 5H), 2.24 (s, 6H), 2.15–2.25 (m, 4H), 1.96–2.17 (m, 2H), ^13^C NMR (DMSO-d6, 400 MHz, δ ppm): 168.4, 154.5, 153.7, 137.5, 136.6, 130.5, 129.3, 125.7, 125.4, 120.0, 63.7, 62.4, 20.8, HREI-MS: m/z calculated for C_26_H_32_BN_3_O_5_ [M]^+^ 477.2544; found 477.2547.

(3,5-dimethylphenyl)methyl 4-[1-(1-hydroxy-3H-2,1-benzoxaborole-6-carbonyl)azetidin-3-yl]piperazine-1-carboxylate 45, Yield: 78%, ^1^H NMR (DMSO-d6, 400 MHz, δ ppm): 9.29 (br.s., 1H), 8.01 (d, *J* = 1.6 Hz, 1H), 7.73 (d, *J* = 7.8 Hz,1H), 7.47 (d, *J* = 7.7 Hz, 1H), 6.88–6.98 (m, 3H), 5.02 (s, 2H), 4.97 (s, 2H), 4.03–4.23 (m, 2H), 3.82–3.90 (m, 2H), 3.36–3.44 (m, 4H), 3.12–3.20 (m, 1H), 2.26–2.34 (m, 4H), 2.24 (s, 6H), ^13^C NMR (DMSO-d6, 400 MHz, δ ppm): 169.3, 156.3, 154.5, 137.4, 136.5, 131.6, 130.1, 129.2, 128.0, 125.4, 124.2, 121.5, 70.0, 66.3, 62.9, 53.6, 48.7, 20.8, HREI-MS: m/z calculated for C_25_H_30_BN_3_O_5_ [M]^+^ 463.2388; found 463.2390.

*(3,5-dimethylphenyl)methyl 4-[1-(1-hydroxy-3H-2,1-benzoxaborole-5-carbonyl)azetidin-3-yl]piperazine-1-carboxylate 46*, Yield: 77%, ^1^H NMR (DMSO-d6, 400 MHz, δ ppm): 9.32 (br.s., 1H), 7.78 (d, *J =* 7.6 Hz, 1H), 7.63 (s, 1H), 7.56 (d, *J =* 7.6 Hz,1H), 6.88–6.98 (m, 3H), 5.00 (s, 2H), 4.97 (s, 2H), 4.26–4.38 (m, 2H), 3.83–3.4.02 (m, 2H), 3.35–3.42 (m, 4H), 3.11–3.18 (m, 1H), 2.25–2.33 (m, 4H), 2.24 (s, 6H), ^13^C NMR (DMSO-d6, 400 MHz, δ ppm): 169.3, 156.3, 154.5, 137.4, 136.5, 131.6, 130.1, 129.2, 128.0, 125.4, 124.2, 121.5, 70.0, 66.3, 62.9, 53.6, 48.7, 20.8 HREI-MS: m/z calculated for C_25_H_30_BN_3_O_5_ [M]^+^ 463.2388; found 463.2391.

*(3,5-dimethoxyphenyl)methyl 4-[1-(1-hydroxy-3H-2,1-benzoxaborole-6-carbonyl)pyrrolidin-3-yl]piperazine-1-carboxylate 47*, Yield: 71%, ^1^H NMR (DMSO-d6, 400 MHz, δ ppm): 9.29 (br.s, 1H), 7.78 (d, *J* = 7.7 Hz, 1H), 7.63 (s, 1H), 7.56 (d, *J* = 7.6 Hz, 1H), 6.38–6.54 (m, 3H), 4.93–5.06 (m, 4H), 4.26–4.40 (m, 4H), 3.83–4.02 (m, 2H),3.73 (s, 6H), 3.35–3.42 (m, 4H), 3.11–3.18 (m, 1H), 2.25–2.33 (m, 4H), ^13^C NMR (DMSO-d6, 400 MHz, δ ppm): 160.6, 129.4, 121.6, 105.6, 99.5, 69.9, 64.0, 55.4, 50.8, HREI-MS: m/z calculated for C_26_H_32_BN_3_O_7_ [M]^+^ 509.2442; found 509.2444.

*(3,5-dimethoxyphenyl)methyl 4-[1-(1-hydroxy-3H-2,1-benzoxaborole-5-carbonyl)pyrrolidin-3-yl]piperazine-1-carboxylate 48*, Yield: 66%, ^1^H NMR (DMSO-d6, 400 MHz, δ ppm): 9.27 (br.s, 1H), 7.78 (d, *J* = 7.4 Hz, 1H), 7.64 (s, 1H), 7.46–7.63 (m, 1H), 6.45–6.53 (m, 2H), 6.43 (s, 1H), 4.93–5.08 (m, 4H), 3.71–3.75 (m, 2H), 3.70 (s,6H), 3.46–3.68 (m, 6H), 3.18–3.29 (m, 1H), 2.33–2.46 (m, 4H), 1.97–2.18 (m, 2H), ^13^C NMR (DMSO-d6, 400 MHz, δ ppm): 160.3, 130.7, 130.3, 125.6, 125.4, 120.0, 106.8, 105.3, 99.3, 55.1, HREI-MS: m/z calculated for C_26_H_32_BN_3_O_7_ [M]^+^ 509.2442; found 509.2444.

*(3,5-dimethoxyphenyl)methyl 4-[1-(1-hydroxy-3H-2,1-benzoxaborole-6-carbonyl)azetidin-3-yl]piperazine-1-carboxylate 49*, Yield: 83%, ^1^H NMR (DMSO-d6, 400 MHz, δ ppm): 9.27 (br.s., 1H), 7.99–8.01 (m, 1H), 7.73 (d, *J =* 8.0 Hz, 1H), 7.47 (d, *J =* 8.0 Hz, 1H), 6.41–6.50 (m, 3H), 5.00 (s, 2H), 4.99 (s, 2H), 4.26–4.33 (m, 4H), 3.84–3.91 (m, 2H), 3.72 (s, 6H), 3.35–3.48 (m, 4H), 3.12–3.20 (m, 1H), 2.25–2.35 (m, 4H), ^13^C NMR (DMSO-d6, 400 MHz, δ ppm): 160.6, 156.4, 154.5, 139.3, 134.3, 131.8, 130.1, 130.0, 129.5, 129.1, 121.5, 105.2, 99.4, 55.2, 54.4, 53.5, HREI-MS: m/z calculated for C_25_H_30_BN_3_O_7_ [M]^+^ 495.2286; found 495.2285.

*((3,5-dimethoxyphenyl)methyl 4-[1-(1-hydroxy-3H-2,1-benzoxaborole-5-carbonyl)azetidin-3-yl]piperazine-1-carboxylate 50*, Yield: 73%, ^1^H NMR (DMSO-d6, 400 MHz, δ ppm): 9.34 (br.s., 1H), 7.79 (d, *J =* 7.4 Hz, 1H), 7.65 (s, 1H), 7.59 (d, *J =* 7.4 Hz, 1H), 6.42–6.51 (m, 3H), 5.00 (s, 2H), 4.99 (s, 2H), 4.23–4.31 (m, 2H), 3.83–3.91 (m, 2H), 3.72 (s, 6H), 3.34–3.46 (m, 4H), 3.14–3.20 (m, 1H), 2.23–2.36 (m, 4H), ^13^C NMR (DMSO-d6, 400 MHz, δ ppm): 169.2, 160.5, 154.3, 139.4, 134.9, 130.6, 126.2, 120.7, 106.7, 105.8, 99.3, 69.9, 66.0, 55.1, 53.6, 48.7, 43.3, HREI-MS: m/z calculated for C_25_H_30_BN_3_O_7_ [M]^+^ 495.2286; found 495.2286.

### 4.3. Molecular Modelling Studies

All visualizations and molecular modelling studies were done using Schrodinger Suite 2018-1 [19]. The co-crystal structure of ATX in complex with the **HA155** boronic acid inhibitor (PDB ID: 2XRG) [9] was used as a template for the docking studies due to the similarity between HA155 and examined molecules. The protein was prepared (modelled) using Protein Preparation Wizard (with Epik v4.3, Impact v7.8, and Prime v5.1) using default settings. Missing hydrogen atoms and sidechains were added, the hydrogen bond network was optimized, and the structure was minimized using the OPLS-2005 force field [38]. The initial neutral 3D structures of ligands were generated by LigPrep and OPLS2005 force-field was used.

Water molecules were removed from the protein structure, and zinc ions of the active site were retained within the structure for docking studies. Docking was performed using Covalent Docking v1.3 [15] within Glide v7.8, where Thr209 of ATX was selected as a reactive residue in the formation of covalent bond in the reaction of boron acid addition [20]. Default values were used for the receptor grid generation around **HA155** ligand in the binding site. Performance of the covalent docking was tested by re-docking of **HA155** into ATX (Figure 5). This verified protocol was used for the docking of boronate compounds in this paper.

### 4.4. Biochemical Enzyme Assay

**Figure 9:**
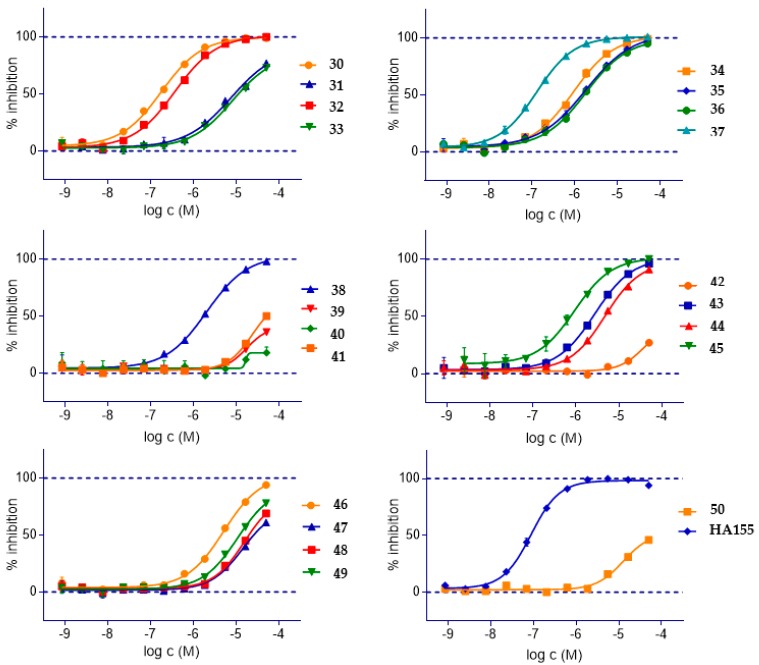
ATX inhibition results of biochemical testing of new benzoxaborole inhibitors. 11—concentrations dose–response curves, as average of duplicates, are presented.

Benzoxaborole derivatives were tested in the choline detection assay for ATX inhibition measuring ATX activity using LysoPC (16:1) as a substrate (Figure 9). Mother plates with serial dilutions of compounds in pure DMSO were prepared from 10 mM DMSO stock solutions. Storplate-384-deep-well-V plates (Perkin Elmer) were also used for mother plate preparation. Compounds were diluted in a DMSO in a ratio of 1:3. An aliquot of 200 nL of compound solution was transferred from the mother plate to the test plate with a Mosquito nanoliter liquid handling system (TTP labtech). The assay was performed in Spectra-Plate-384 TC, transparent, (Perkin Elmer, Cat#6007658) using a 20 µL assay volume. The total final percentage of DMSO in the test media was 1%. Compounds were tested at 11 consecutive three-fold dilutions starting from 50 µM (final concentrations) in duplicate, covering range of 0.0008–50 μM to determine IC_50_ values. The control compound (**HA 155**) was tested on every test plate. After dispensing of compounds directly to the dry plate, we added 10 μL of recombinant ATX (2 ng/mL) in a Tris–HCl buffer (140 mM NaCl, 1mM MgCl_2_, 5 mM KCl, 3 mM CaCl_2_, 1 mg/ml bovin serum albumin (BSA, fatty acid free) and 100 mM Tris–HCl, pH 9). Finally, 10 μL of 200 μM LysoPC (16:1) in a Tris–HCl buffer was added to each well, while the plate was incubated 90 min at 37 °C. The above-described solution with dimethyl sulfoxide but without the test compound was used as a control. LysoPC without ATX was used as a control for the autohydrolysis of LysoPC. After 1.5 h of incubation, 20 μL of stop solution and developer was added {Choline oxidase (1.2 U/mL), Peroxidase (1.2 U/mL), TOOS (2.7 mM), 4-Aminoantipyrine (2.7 mM), and EDTA (50 mM)} to the reaction mixture, and absorbance was measured after 30 min of incubation. The absorbance was measured by using Perkin-Elmer EnVision plate reader (λ = 555 nm). Data were analyzed using GraphPad Prism software. The calculation of IC_50_ data, curves and quality control (QC) analysis was made by using Excel tools and GraphPadPrism software. Briefly, individual concentration–effect curves were generated by plotting the logarithm of the tested concentration of tested compounds (X) vs. corresponding percentage inhibition values (Y) using a least squares (ordinary) fit. Best fit IC_50_ values were calculated using the Log (inhibitor) vs. response – variable slope (four parameters) equation, where Y = 100/(1 + 10^((LogIC_50_–X)*HillSlope)). QC criteria parameters (Z,’ S:B, ΔmP) were checked for every plate.

### 4.5. Lipophilicity Determination

Chrom logD values were determined from the following equation: Chrom logD = 0.0857 × CHI − 2 [39]. Chromatographic hydrophobic index (CHI) values were determined from the gradient retention times (RT). These values approximately corresponded to the volume percentage of organic component in the mobile phase when the compound elutes. CHI values were determined at pH 7.4. Chromatograms were obtained using on Agilent 1100 Series liquid chromatography system with an HPLC diode-array detector (DAD) coupled with a Micromass Quattro micro atmosphere pressure ionization (API) mass spectrometer. The column used for analyses was Phenomenex Luna C18 (50 × 3 mm, 5 µm, 100 Å). The column was maintained at 25 °C. The flow rate was 1.0 mL/min. The mobile phase was composed of 50 mM ammonium acetate in water at pH 7.4 (solvent A) and acetonitrile (solvent B). The solvent gradient condition was initially 0.5% B, and then it increased linearly to 100% B over 3.0 min, remaining at 100% B for 0.5 min, then decreasing linearly to 0% B over 0.2 min followed by an equilibration period of 1.3 min prior to the next injection. The total experimental duration was 5 min. Injection volume was 2.0 µl. Samples temperature was maintained at 15 °C in the autosampler chamber. A group of ten standard compounds having different CHI values was used for calibration of RP-HPLC [40].

### 4.6. Kinetic Solubility

Turbidimetric solubility allows for a rapid determination of kinetic solubility by using small amounts of the test compound. Assay controls were α-naphtoflavone (low solubility) and sulfaphenazole (expected solubility >100µM). DMSO stock solutions (10 mM) of each compound were first serially diluted 1 in 3 in DMSO and then spiked into an aqueous buffer (100 mM PBS), resulting in 5 following final concentration: 100, 30, 10, 3 and 1 µM (each concentration was tested in duplicate). The prepared solutions were then incubated at 37 °C for 2 h with gentle shaking, followed by absorbance measurements on microplate reader (Infinite F500, Tecan, CH) at 620 nm (at t = 0 and t = 2h). Absorbance is proportionally increased with the concentration of insoluble particles as compound precipitates. Compound samples were compared to a solvent control in an aqueous buffer (DMSO 1% final concentration), and a significant increase of sample absorbance was considered to have occurred when the absorbance reached a 3-fold standard deviation of the average DMSO absorbance. The calculation of solubility range data was made by Excel tool software. Results are expressed as an estimated solubility range.

### 4.7. Plasma Stability

The in vitro plasma stability of the test compounds was studied in human and rat plasma with propranolol, benflorex, and eucatropine as control substances. All compounds at final concentrations of 5 μM (0.5% DMSO) were incubated in a phosphate buffer (50 mM, pH 7.4) for 4 h at 37 °C together with plasma. Aliquots were taken at different time points (0, 30, 120 min and 4 h), followed by reaction termination by the addition of CH_3_CN:CH_3_OH 2:1 v/v., which contained diclofenac and warfarin as internal standards (IS). Samples were then centrifuged (at 1600 rpm at 4 °C for 30 min), and the resulting supernatants were subjected to LC/MS/MS analysis. Results are expressed as % remaining.

### 4.8. In Vitro Metabolic Stability in Rat and Liver Human Microsomes

Metabolic stability was assessed in rat and human liver microsomes, with testosterone and propranolol as positive controls. All compounds, at a final concentration of 1 μM (0.03% DMSO), were incubated in a phosphate buffer (50 mM, pH 7.4) for 60 min at 37 °C, together with liver microsomes in the absence and presence of NADPH generating system (nicotinamide adenine dinucleotide phosphate (NADP, 2 mM), glucose-6-phosphate (20 mM), glucose-6-phosphate dehydrogenase (6U) and magnesium chloride (2 mM)). Aliquots were taken at different time points (0, 10, 20, 30, 45 and 60 min), followed by reaction termination by addition of CH_3_CN:CH_3_OH 2:1 *v/v*, containing diclofenac as internal standard (IS). Samples were then centrifuged (at 2000 rpm at 4 °C for 30 min), and resulting supernatants were subjected to LC/MS/MS analysis. The in vitro half-life was calculated in GraphPadPrism software from % remaining vs. time regression using non-linear regression fit. In vitro intrinsic clearance, expressed as mL/min/g liver, was determined from in vitro half-life and normalized for the protein concentration in the incubation mixture, assuming a 52.5 mg/g liver. Finally, predicted in vivo hepatic clearance, expressed as %LBF (liver blood flow), was determined from in vitro intrinsic clearance normalized for the weight over whole body weight (87.5 g/kg for mouse and 25.7 g/kg for human) and liver blood flow (131 mL/min/kg for mouse and 21 mL/min/kg for human).

### 4.9. Plasma Protein Binding Assay

Plasma protein binding was measured using the equilibrium dialysis technique. Positive controls included in this assay were: Acebutolol (low binding) and nicardipine (very high binding). The assay was performed in a 96-well Teflon dialysis unit, where each well consisted of two chambers separated by a dialysis membrane. Plasma (human and rat, commercially obtained from Seralab) spiked with each compound (final concentration: 5 µM, 0.5% DMSO) were added to one chamber, and a blank buffer solution (60 mM Na_2_HPO_4_/14 mM KH_2_PO_4_/70 mM NaCl; pH 7.4) was added to the other side of the well. Each compound was analyzed in duplicate, for 4 h at 37 °C. After 4 h, both the plasma (free and bound fraction) and buffer (only free fraction) side of the well were sampled, matrix matched, then precipitated with a STOP solution (CH_3_CN:CH_3_OH 2:1 v/v, containing diclofenac as internal standard), and centrifuged at 2000 rpm for 30 min (+4 °C). The resulting supernatants were analyzed by LC-MS/MS. The fraction bound was calculated as the concentration difference (analyte vs. internal standard peak area ratio) in the plasma and buffer sides divided by the total concentration in the plasma side. Sampling was also performed from spiked plasma at t = 0 min in order to determine recovery, i.e., % of compound detected at t = 4 h in both the plasma and buffer chambers divided by the compound detected at t = 0 min.

## 5. Conclusions

Our strategy was to target the Thr209 and zinc atoms in the binding pocket of ATX, with the boronic acid isostere: Borinic acid. As well as similar enzyme potencies, we expected better ADME properties of such modified compounds. The group occupying the hydrophobic lipid-binding pocket was optimally that of the Pfizer molecule **PF-8380**. As the core spacer, we used saturated bicyclic systems (piperazino–piperidine, piperazino–pyrolidine and piperazino azetidine). This overall design was ratified by docking experiments, and the molecules were then synthesized (compounds **30–50**). ATX inhibition in vitro and ADME properties were measured aiming for better overall profile than that possessed by **HA130** and **HA155**. The compounds **HA130** and **HA155** were reported to be active in the in vivo study, lowering LPA significantly but with a very short half-life (less than five min) [10]. The four new compounds designed, synthesized and tested in this work (**30, 32, 34,** and **37**) showed ATX inhibition in the nanomolar range with the IC_50_ value for the most active compound **37** being 130 nM, which is only two times less potent than **HA155**. Compound **37** has a core spacer more rigid than in **HA155**, designed for better stability. Most of ADME parameters for tested compounds were better than for **HA155**. The plasma stability for compound **37** showed 71.2% and 79.1% remaining in rat and human plasma, respectively, after four hours. **HA155** showed 65.7% and 42.9% in rat and human plasma, respectively, also measured at four hours. Furthermore, the PPB of **37** was significantly lower than for **HA155** (fraction unbound for **37** was 1.6% in rat and 2.1% in human plasma, while for **HA155** was 0.4% and less than 0.1%, respectively). Based on our findings, we have identified potent ATX inhibitors with in vitro enzyme inhibition potencies similar to that of **HA155** but with an improved ADME profile.

Based on these findings, additional experiments in vivo are ongoing to further evaluate the PK properties of the compound **37** head to head with **HA155**. If the PK data of compound **37** show better stability and half-life then **HA155**, the compound **37** will be tested in relevant animal oncology models.

## Figures and Tables

**Table 1:** Structures, inhibitory activities, calculated logP and determined Chrom logD values.

Compound	Structure			^a^ IC50 Value (µM)	^b^ clog P	^c^ Chrom logD
	Warhead	Core	Lipophilic Tail			
	W	CS	R			
**30**	W 1	CS 1	Cl	0.18 ± 0.04	3.82	4.52
**31**	W 4	CS 1	Cl	7.5 ± 5.60	3.61	4.63
**32**	W 2	CS 1	Cl	0.37± 0.35	3.82	4.38
**33**	W 3	CS 1	Cl	8.0 ± 2.58	3.61	5.40
**34**	W 1	CS 2	Cl	0.92 ± 0.82	4.02	5.49
**35**	W 2	CS 2	Cl	1.8 ± 1.75	4.02	4.58
**36**	W 4	CS 2	Cl	1.8 ± 1.52	3.99	4.58
**37**	W 1	CS 3	Cl	0.13 ± 0.12	3.60	4.58
**38**	W 2	CS 3	Cl	2.0 ± 1.73	3.60	4.46
**39**	W 1	CS 1	H	>50 ± 4.20	2.75	3.05
**40**	W 2	CS 1	H	>50 ± 3.08	2.75	2.90
**41**	W 1	CS 3	H	23 ± 1.18	2.40	3.07
**42**	W 2	CS 3	H	>50 ± 32.84	2.40	2.90
**43**	W 1	CS 1	CH_3_	2.7 ± 2.11	3.17	4.13
**44**	W 2	CS 1	CH_3_	4.7 ± 1.14	3.17	3.99
**45**	W 1	CS 3	CH_3_	1.0 ± 1.15	2.90	4.17
**46**	W 2	CS 3	CH_3_	5.1 ± 2.75	2.90	4.01
**47**	W 1	CS 1	OCH_3_	14 ± 5.50	2.51	3.19
**48**	W 2	CS 1	OCH_3_	15 ±6.07	2.51	3.03
**49**	W 1	CS 3	OCH_3_	11 ± 4.59	2.48	3.21
**50**	W 2	CS 3	OCH_3_	13 ± 3.26	2.48	3.07
**HA155**	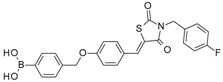			0.088 ± 0.04 0.0057 ^d^	3.76	NA

^a^ IC_50_ values determined in choline release assay using LPC C16:0 as a substrate. The results are shown as the mean standard deviation from duplicate experiments. ^b^ calculated log P was the octanol/water partition coefficient calculated using Percepta. ^c^ Chrom logD was determined at pH 7.4 using Luna C18 (50 × 3 mm i.d., 5 µm) column. ^d^ IC_50_ values taken from literature [26].

**Table 2:** In vitro ADME profile of selected compounds.

Compound	^a^ Solubility	^b^ PS (rat/human)	^c^ LMS (rat/human)	^d^ PPB (rat/human)
30	>100	64.5/66.5	86/82	98.8/98.4
31	>100	67.9/71.6	86/85	NA ^e^/97.8
34	>100	61.6/70.6	77/89	NA ^e^/99.2
35	>100	63.0/59.2	86/89	NA ^e^/98.3
37	30–100	72.2/79.1	62/85	98.4/97.9
38	30–100	60.1/79.1	82/79	NA ^e^
43	>100	59.5/79.2	80/85	96.1/97.8
45	>100	68.6/72.8	61/84	94.4/95.9
HA155	3–10 ^f^ 6.8	65.7/42.9	<30/54	99.9/>99.9

^a^ Kinetic solubility range after 1h [µM]. ^b^ Plasma stability after 4 h [%remaining]. ^c^ LMS: Metabolic stability in human/rat liver microsomes -> predicted in vivo hepatic clearance expressed as %LBF. ^d^ PPB: Plasma protein binding in human/rat -> fraction bound [%]. ^e^ recovery issue. ^f^ solubility value taken from literature [7].
